# Efficacy of uniportal versus multiportal video-assisted thoracoscopic lobectomy for non-small cell lung cancer: A retrospective analysis

**DOI:** 10.12669/pjms.40.6.9313

**Published:** 2024-07

**Authors:** Xing Zheng, Wenmin Wang, Xiang Li, Pengyixiang He, Xu Wu

**Affiliations:** 1 Xing Zheng, Department of Thoracic Surgery, The First School of Clinical Medicine, Southern Medical University, Guangzhou, Guangdong Province 510515, China; 2 Wenmin Wang Department of Breast Surgery, Nanfang Hospital Baiyun Branch, Guangzhou, Guangdong province 510405, China; 3 Xiang Li Department of Emergency, Nanfang Hospital, Southern Medical University, Guangzhou, Guangdong Province 510515, China; 4 Pengyixiang He, Department of Thoracic Surgery, The First School of Clinical Medicine, Southern Medical University, Guangzhou, Guangdong Province 510515, China; 5 Xu Wu, Department of Thoracic Surgery, The First School of Clinical Medicine, Southern Medical University, Guangzhou, Guangdong Province 510515, China

**Keywords:** Non-small-cell lung cancer, Video-assisted thoracoscopic surgery, Uniportal, Multiportal

## Abstract

**Objective:**

To compare the uniportal and multiportal video-assisted thoracoscopic surgery (VATS) in patients with non-small-cell lung cancer (NSCLC).

**Methods:**

Medical records of 128 patients with NSCLC who underwent surgical treatment in the First School of Clinical Medicine, Southern Medical University from August 2020 to February 2022 were retrospectively analyzed. There were 60 patients who underwent uniportal VATS (UVATS group) and 68 patients underwent multiportal VATS (MVATS group). The relevant indexes, complications, postoperative pain levels and quality of life, recurrence, metastases and survival between the two groups were compared.

**Results:**

UVATS was associated with longer operation time and higher intraoperative blood loss compared to MVATS (*P*<0.05). The postoperative drainage volume, and the visual analogue scale (VAS) scores at 24 and 72 hours were lower in the UVATS group compared to the MVATS group, while the chest tube retention time and hospitalization time were shorter than those in the MVATS group (*P*<0.05). The quality of life at six months after surgery in the UVATS group was significantly higher than that in the MVATS group (*P*<0.05).

**Conclusions:**

UVATS and MVATS have similar outcomes in patients with NSCLC. Although UVATS surgery takes longer and is associated with more interoperative bleeding, it can reduce postoperative pain, shorten postoperative recovery time, and help further improve the quality of life of patients after surgery.

## INTRODUCTION

Lung cancer is the leading cause of cancer-related deaths worldwide, and non-small-cell lung cancer (NSCLC) accounts for more than 80% of all lung cancer cases.^1^,^2^ At present, surgery is the first treatment for patients with NSCLC.^2^,^3^ However, open chest surgery is associated with significant trauma and many complications, that negatively impact patient’s quality of life.^3^,^4^

In recent years, with the development of endoscopic surgery technology, thoracoscopy gradually became a method of choice in the treatment of NSCLC.^5^ Multiportal video-assisted thoracoscopic surgery (MVATS) is a relatively classic surgical technique performed through several puncture holes in the thoracic wall, which allows to clearly visualize the lesions, and to perform a precise resection with minimal trauma and fast postoperative recovery.^6^,^7^ In 2010, the first uniportal video-assisted thoracoscopic surgery (UVATS) was performed. It requires a single puncture hole in the fourth or fifth intercostal space at the front of the axilla, through which the thoracoscopy lens and surgical instruments are inserted into the chest cavity. Studies have shown that while UVATS can further reduce the surgical trauma and postoperative pain,^7^,^8^ the use of a single incision may interfere with the entry and exit of surgical instrument, prolong the operation time, and increase the difficulty of lymph node dissection.^9^

At present, there is still no consensus on the advantages of either techniques in the treatment of lung cancer.^10^ Studies have focused on operation time, intraoperative blood loss, postoperative hospitalization, and complications, but few study on postoperative quality of life. In recent years, our hospital has successfully used both UVATS and MVATS approaches for NSCLC surgery. The aim of the current retrospective study was to further compare the efficacy of UVATS and MVATS in treating patients with NSCLC from the perspective of perioperative and postoperative indicators.

## METHODS

Medical records of 128 patients with NSCLC who underwent VATS in the First School of Clinical Medicine, Southern Medical University from August 2020 to February 2022, were retrospectively analyzed. Patients were divided into UVATS and MVATS groups according to the type of surgical incision used during the VATS procedure.

### Inclusion criteria:

- Diagnosed of NSCLC confirmed by pathological test;^11^
- TNM staging of stage I to stage II;
- Preoperative confirmation of the absence of metastatic lesions;
- Clear indications for surgery;
- Complete medical records.

### Exclusion criteria:

- Preoperative chemotherapy and radiation therapy;
- Severe pleural adhesions;
- Patients with cardiopulmonary insufficiency;
- History of chest surgery;
- Transition to open chest surgery midway.

### Ethical Approval

All procedures involving human participants were in accordance with the ethical standards of the institutional and/or national research committee(s) and with the Helsinki Declaration (as revised in 2013). Written informed consent was obtained from the patient or legal guardian, and the medical ethics Committee of our hospital approved this study (No. 2023062801, Date: June 28, 2023).

### Surgical methods

After general anesthesia, patient’s position was adjusted to the unaffected side position. One-lung ventilation on the healthy side was established, and chest pad was placed to elevate the chest. The intercostal space on the affected side was maximized and the tissue was disinfected. The surgical procedures in both groups were performed by the same team.

### UVATS^12^

Based on the tumor site, an incision was made in the fourth intercostal space from the anterior axillary line to the midaxillary line in the right upper lung. If the tumor was located in another location, an incision was made in the 5^th^ intercostal space. The incision size was approximately 3.5cm. The subcutaneous tissue was cut using the electric knife, and the incision protective sleeve was placed. Thoracoscope was inserted and the thorax was explored to determine the tumor location. Pulmonary veins, arteries, and bronchi were separated. Pulmonary fissures were separated by using a disposable cutting and suturing device, and the lung lobe tissue was removed. Routine thoracic lymph node dissection was performed, bleeding stopped, and the chest cavity flushed. After performing a lung drum examination and confirming no air leakage, a thoracic drainage tube was inserted, and the incision was closed.

### MVATS^13^

The first incision was made between the 7th or 8th ribs, with a size of approximately 1.5cm (the observation hole), and a trocar of 10 mm was inserted. The second incision was made between the 8th rib, with a size of 1-2cm (auxiliary operating hole). The third incision was performed between the 4th rib at the front of the axilla, with a size of 3-4 cm (the main operating hole). A cut protection sleeve was used, and lung lobectomy and lymph node dissection were performed similarly to UVATS procedure. After completion, a drainage tube was inserted, the incision was closed, and the surgery was completed.

### Observation indicators:

### Perioperative indicators

operation time, intraoperative blood loss, postoperative drainage volume, postoperative pain at 24 and 72 hours, chest tube indwelling time, and hospital stay. Pain level was evaluated using the visual analog scale (VAS), with a score ranging from 0 (no pain) to 10 (unbearable pain).^14^

### Complications

Atelectasis, pleural effusion, secondary hemostasis, bronchopleural fistula, and chylothorax.

### Quality of life

It was evaluated using the Functional Assessment of Cancer Therapy-Lung (FACT-L) scale.^15^ The scale includes five dimensions: physical condition, social/family condition, psychological condition, functional condition, and lung cancer specific modules, with seven, seven, six, seven and nine items respectively. Each item is scored 0-4 points, with a total score of 0-144 points. Higher score indicates better quality of life.

Recurrence, metastasis, and survival 12 months after surgery.

### Statistical analysis

Statistical analysis was conducted using SPSS22.0 software. Normally distributed measurement data were expressed as (*χ̅*±*S*). Comparison between the groups was done by independent sample *t* test, and the comparison within the group was done by the paired *t* test. Non-normally distributed data were presented by the median interquartile range (IQR). Mann Whitney *U* test was used for comparison between the two groups. Counting data were expressed as n(%), and Chi-squared test was used for comparison between the groups. *P*<0.05 indicated that the difference is statistically significant.

## RESULTS

Medical data of 128 patients (79 males and 49 females) were included in this study. Age ranged from 46 to 78 years, with a mean age of 64.0 ± 6.7 years. In terms of TNM staging, 71 patients had stage I and 57 had stage II. There were 60 patients in the UVATS group and 68 patients in the MVATS group. Baseline data were similar in both groups (*P*>0.05) (Table-I). Patients in the UVATS group had longer operation time and more intraoperative blood loss compared to the MVATS group (*P*<0.05). The postoperative drainage volume, VAS scores at 24 and 72 hours in the UVATS group were lower than those in the MVATS group, and the duration of thoracic tube retention and hospitalization was shorter than that in the MVATS group (*P*<0.05) (Table-II). There was no difference in the rate of complications between the UVATS (5.0%) and the MVATS groups (5.9%) (*P*>0.05) (Table-III). There was no statistically significant difference in the preoperative FACT-L scores between the two groups (*P*>0.05). At six months after surgery, the FACT-L scores of both groups improved compared to pre-surgery score, were significantly higher in the UVATS group (*P*<0.05) (Table-IV). Twelve months after the surgery, there was one case of recurrence in the UVATS group and two cases of recurrence in the MVATS group. There were no deaths in either group. Overall, there was no statistically significant difference in the recurrence rate and mortality rate between the two groups (*P*>0.05).

**Table-I:** Comparison of general data between the two groups.

Group	n	Gender (Male/Female)	Age (Year)	TNM stage (n)		Tumor location (n)				
	
				= 1 \* ROMAN \* MERGEFORMAT I	= 2 \* ROMAN \* MERGEFORMAT II	Right upper lobe	Right middle lobe	Right lower lobe	Left superior lobe	Left inferior lobe
UVATS group	60	35/25	64.1±5.9	37(61.7)	23(38.3)	13(21.7)	9(15.0)	10(16.7)	19(31.6)	9(15.0)
MVATS group	68	44/24	63.8±7.5	38(55.9)	30(44.1)	15(22.1)	10(14.7)	13(19.1)	16(23.5)	14(20.6)
*χ^2^*/*t*		0.548	0.256	0.440		1.437				
*P*		0.459	0.799	0.507		0.838				

**Table-II:** Comparison of surgical indicators between the two groups.

Group (n)	Operation time (minute)	Intraoperative blood loss (ml)	Postoperative drainage volume (ml)	Postoperative 24 hours VAS score (score)	Postoperative 72 hours VAS score (score)	Chest tube retention time (day)	Hospital stay (day)
UVATS group (n=60)	184±13	171±148	908±58	4(3.5,5)	3(3,4)	5(4,6)	9(9,10)
MVATS group (n=68)	143±12	126±11	943±54	5(5,6)	4(3,4)	6(5,7)	11.5(10,12)
*t/z*	18.295	20.896	-3.553	-4.014	-2.309	-3.842	-6.84
*P*	<0.001	<0.001	0.001	<0.001	0.021	<0.001	<0.001

**Table-III:** Comparison of complications between the two groups.

Group	n	Complication					Total

		Atelectasis of lung	Pleural effusion	Recurrent hemostasis surgery	Bronchopleural fistula	Chylothorax	
UVATS group	60	1 (1.67)	1 (1.67)	0 (0.00)	1 (1.67)	0 (0.00)	3 (5.0)
MVATS group	68	1 (1.47)	1 (1.47)	1 (1.47)	0 (0.00)	1 (1.47)	4 (5.9)
Continuity-corrected χ^2^	-						0.000
P	-						1.000

**Table-IV:** Comparison of quality of life between the two groups.

Group (n)	Preoperative	Six months after surgery	t	P
UVATS group (n=60)	75.82±5.3	106.2±8.2	-30.579	<0.001
MVATS group (n=68)	75.5±6.1	97.7±8.0	-17.486	<0.001
*t*	0.311	5.970	-	-
*P*	0.757	<0.001	-	-

## DISCUSSION

The results of this study show that the efficacy of UVATS and MVATS in the treatment of NSCLC is comparable. UVATS appears to be more effective in reducing postoperative pain, shortening postoperative recovery time, and further improving quality of life of the patients, which is consistent with the findings by Lee et al.^12^ and Li et al.^13^

At present, the main surgical method for early lung cancer is to remove the diseased lung lobe. When performing this surgery, traditional chest VATS requires three puncture holes.^16^ Qian et al.^17^ compared the therapeutic effects of MVATS with traditional thoracotomy surgery in elderly patients with lung cancer, and showed that MVATS had lower inflammatory response and less impact on the body’s immune function.

Recently, with the development of the concept of rapid rehabilitation surgery, there is a gradual increase in the use of UVATS that requires only one incision.^18^ Studies have shown that this surgical method has more advantages in reducing surgical trauma and postoperative pain.^2^,^18^-^21^ Kapicibasi et al.^2^ have shown that using UVATS not only allows to safely perform lung biopsy and resection, but is also safe for bullectomy. Zhang J et al.^20^ also reported that patients who undergo UVATS have less trauma, which is beneficial for lung function and postoperative recovery. In addition, the number of lymph nodes that is removed during UVATS is comparable to that of MVATS. Similarly, Mizukami et al.^21^ indicated that single-port VATS pulmonary wedge resection offers better pain control and is more cost-effectiveness compared to the three-port VATS pulmonary wedge resection. UVATS requires a single puncture hole, with a smaller chest wound involving only one intercostal space, which can reduce the trauma to muscles and nerves that is caused by the surgery. Subsequently, it reduces the degree of postoperative pain, promotes early mobilization of patients, accelerates postoperative recovery time, and thus shortens postoperative recovery time.^20^-^23^ In terms of surgical complications, we found no significant difference between UVATS and MVATS, which is consistent with the research results of Yao J et al.^24^ Our hospital has also strengthened rapid surgical intervention for patients with NSCLC during the perioperative period, thus using various methods to more effectively reduce surgical trauma.

In this study, the FACT-L score of the UVATS group at six months after surgery was significantly higher than that of the MVATS group, which is consistent with the study by Cao M et al.^23^, suggesting that UVATS is more helpful in improving postoperative quality of life of patients.

Despite numerous advantages, it is important to emphasize that UVATS is associated with certain difficulties compared to MVATS. It may be challenging to perform various surgical procedures through a 3-5cm incision, which may result in prolonged operation time. In addition, if there is serious accidental blood loss during operation, it may be necessary to switch to thoracotomy, leading to increased patient pain.^22^,^25^ Based on this, we suggest that when choosing to treat patients with NSCLC through VATS in clinical practice, the single hole or three hole pathway should be selected based on the actual situation of the hospital and the patient, to ensure patient safety to the greatest extent. We propose a gradual transition process, first to single puncture hole surgery, and then from large-incision single hole to small-incision single hole. This will ensure the maximal proficiency of the operating team in safely performing UVATS.

### Limitations

The study is retrospective with a sample size of only 128 patients. Moreover, the postoperative observation time was relatively short and no additional objective observation indicators have been included, which may affect the comprehensiveness and objectivity of our conclusions.

## CONCLUSION

UVATS can further reduce surgical trauma, reduce postoperative pain, shorten postoperative recovery time, and help improve postoperative quality of life of patients with NSCLC who undergo minimally invasive thoracic surgery.

### Authors’ contributions:

**XZ** conceived and designed the study.

**WW, XL, PH and XW** collected the data and performed the analysis.

**XZ** was involved in the writing of the manuscript and is responsible for the integrity of the study.

All authors have read and approved the final manuscript.
